# P80 - An evaluation of the efficacy and safety of a dust mite sublingual immunotherapy in allergic asthmatic children: a retrospective clinical data analysis over a three-year period

**DOI:** 10.1186/2045-7022-4-S1-P135

**Published:** 2014-02-28

**Authors:** Emad Arafa, Graham Roberts

**Affiliations:** 1 NMC Specialty Hospital, Dubai, United Arab Emirates; 2 University of Southampton, Southampton, United Kingdom

## Background

A promising treatment for allergic asthma (AA) in children is the sublingual specific immunotherapy (SLIT) which targets mild or moderate persistent AA types. There have not been any studies to investigate the efficacy and safety of adding SLIT to combination therapy (budesonide / formoterol) in asthmatic children sensitive to house dust mite (HDM).

## Aims and objectives

The aims of this current study were to evaluate the effectiveness and safety of adding SLIT to combination therapy (budesonide / formoterol) in HDM sensitive children with AA over a three-year period. Another aim of this study was to evaluate the possibility of reducing the dose of the combination therapy on using SLIT.

## Methods

This study has followed a retrospective clinical and laboratory data analysis. The data were classified according to the treatment protocol given into two groups, both of which continued their treatment plan for a period of three years. Fifty children (Control group) who were treated with combination therapy (budesonide 80 mg /formoterol 4 mcg), two puffs twice daily. The Control group compared to fifty children (SLIT group) who were treated with the same combination therapy plus standardised extract 50/50 Dp / Df, a dust mite SLIT. The data analyses were conducted at baseline and after three years in each and in between groups.

## Results

The results of this study showed that SLIT group has a highly statistically significant reduction in the use of daily doses of the combination treatment (budesonide / formoterol) (p<0.0001), in comparison to control group (p=0.397). In addition, in the SLIT group there were 97% of the patients totally stopped both control and reliever medications in the final six months and only continued with SLIT. Moreover, there were more improvements in FVC and FEV1 in SLIT compared to control group. Additionally, these results revealed that SLIT significantly reduced the clinical outcome scores. There were no systematic (anaphylaxis) side effects reported with SLIT.

## Conclusions

The results of this study suggest that SLIT has a significant role in controlling AA due to dust mite in children. Besides, the results suggest that SLIT is a safe medication for children with AA. Caution must be applied when using the results of this study in practice due to the retrospective design of this study, which means that the findings may not be transferable to routine clinical practice.

